# Interaction Between Polycarboxylate Superplasticizer and Clay in Cement and Its Sensitivity Inhibition Mechanism: A Review

**DOI:** 10.3390/ma18112662

**Published:** 2025-06-05

**Authors:** Yu Gao, Yingying Liu, Guanqi Wang, Jiale Liu, Zijian Cao, Qiwen Yong, Hongwei Zhao

**Affiliations:** 1 School of Material Science and Engineering, Hunan University of Science and Technology, Xiangtan 411201, China; 2 College of Chemistry and Chemical Engineering, China West Normal University, Nanchong 637009, China

**Keywords:** polycarboxylate superplasticizer, clay sensitivity, cement, sacrificial agent

## Abstract

In contemporary construction practices, polycarboxylate superplasticizers (PCEs) have gained extensive utilization in concrete formulation owing to their exceptional dispersive properties and superior water reduction capabilities. Nevertheless, these admixtures demonstrate pronounced susceptibility to clay contamination, a critical limitation that substantially constrains their practical implementation. To mitigate this detrimental effect, multiple technical strategies have been developed to suppress clay sensitivity, with predominant approaches focusing on molecular structure optimization and incorporation of supplementary admixtures. This review systematically investigates the competitive adsorption mechanisms operating at the cement–clay interface. Through rigorous analysis of molecular architecture characteristics and synergistic admixture combinations, we comprehensively review current methodologies for enhancing the clay resistance of PCE-based systems. Furthermore, this paper proposes prospective directions for synthesizing clay-tolerant PCE derivatives, emphasizing molecular design principles and advanced formulation protocols that may inform future research trajectories in construction materials science.

## 1. Introduction

The persistent growth of the global construction sector has led to a corresponding surge in concrete demand. At the same time, there are also increasing demands on concrete properties [1,2,3]. The system of concrete incorporates four principal phases: cementitious binders (typically ordinary Portland cement), mineral aggregates, hydration water, and performance-modifying chemical admixtures [4]. PCEs have become essential admixtures in modern cementitious systems owing to their unique combination of high dispersion efficiency, outstanding water-reducing performance, tunable molecular structures, and enhanced environmental sustainability [5,6,7]. The overexploitation of natural sand and gravel reserves has led to severe depletion of high-quality aggregate resources, resulting in increased clay content in remaining sand and stone deposits [8]. As deleterious components, clay minerals exhibit competitive adsorption behavior with cement particles. PCEs undergo preferential adsorption and intercalation onto clay minerals through physical and chemical effects [9], resulting in significant deterioration of PCE’s functional performance, including concrete fluidity, slump retention, and water-reducing efficiency, consequently increasing concrete production costs [10].

In light of these adverse impacts, significant research has been devoted to understanding clay sensitivity mechanisms and developing effective countermeasures. The most immediate mitigation strategy involves increasing PCE dosage. However, this approach substantially elevates production costs to achieve target performance, rendering it economically impractical for large-scale applications. The second method focuses on modifying the molecular structure of PCEs. Experimental evidence indicates that strategic optimization of side-chain architecture, including length and grafting density, significantly suppresses PCE intercalation in clay interlayer spaces [11]. The introduction of large volume groups such as β-cyclodextrin in the side chain can enhance the steric hindrance caused by PCE which further inhibits the clay sensitivity of PCE [12]. In addition, the loss of the polyethylene oxide (PEO) side chain can be reduced by introducing anions and cations into the main chain [13,14]. Furthermore, topological optimization of PCEs molecules can simultaneously decrease molecular consumption while enhancing steric hindrance effects. Beyond intrinsic molecular structure modification, contemporary strategies incorporate functional admixtures, such as clay-swelling inhibitors and sacrificial agents, to mitigate clay-induced performance degradation [15,16]. The incorporation of these functional admixtures effectively suppress PCE–clay interactions through multiple mechanistic pathways, consequently optimizing the performance of PCEs.

While substantial progress has been made in clay sensitivity mitigation research, translational applications continue to encounter formidable obstacles, primarily associated with economic viability and synthetic complexity. Building upon these foundations, this paper aims to systematically summarize the methods to suppress the sensitivity of clay by exploring the interaction mechanism between clay and PCEs, and identifies promising research directions for overcoming the clay sensitivity of PCEs.

## 2. Polycarboxylate Superplasticizer

### 2.1. Main Types of PCE

Based on monomer structural classification, PCEs can be categorized into five primary types.

- MPEG-PCE

The synthesis of this PCE type predominantly utilizes methoxy polyethylene glycol (MPEG) as the monomer, with two principal synthetic routes available [17]. One method to synthesize is grafting reactions, where the MPEG is grafted into the main chain of poly (methacrylic acid) at 150 °C in a vacuum environment [18]. The second method involves free-radical copolymerization, in which MPEG reacts with monomers such as methacrylic acid in the presence of an initiator at 80 °C [19]. This type of PCE still dominates the European market currently [20]. Werani et al. systematically investigated the clay sensitivity of MPEG-PCEs with varying side-chain lengths. The results demonstrated that MPEG-PCEs containing longer side chains (45 ethylene oxide units) displayed significantly higher clay sensitivity compared to counterparts with shorter side chains (23 ethylene oxide units) [21]. The MPEG-PCE with longer side chains required a dosage of 0.71% bwoc to achieve a paste spread of 26 cm, whereas the shorter-chain analogue attained comparable workability (26 cm spread) at only 0.35% bwoc.

- APEG-PCE

This type of PCE is based on the allyl polyethylene glycol (APEG) macromonomer, which is a three-carbon macromonomer [22]. One synthesis method is the traditional bulk polymerization reaction. Maleic anhydride (MA) and APEG are reacted in a laboratory flask under solvent-free conditions and there is no solvent present during the reaction process [23]. This method enables the synthesis of conventional APEG-PCEs featuring an alternating copolymer structure of MA and APEG units. Another way is free-radical copolymerization [24]. AA serves as an alternative free-radical copolymerization monomer to MA when reacting with APEG. This approach enables the creation of diverse molecular architectures, significantly enhancing the workability performance of APEG-PCE compared to conventional bulk polymerization methods [25]. Furthermore, Lei et al. demonstrated that APEG-PCEs possessing short side chains (n_EO_ = 7) were found to exhibit lower clay sensitivity as compared to that of conventional MPEG-PCEs holding long pendant chains (n_EO_ = 45) [26]. The saturated adsorption capacity of APEG-PCEs on clay surfaces ranged from 110 to 170 mg/g, whereas MPEG-PCE exhibited a significantly higher capacity of 241.5 mg/g—approximately double the maximum value observed for APEG-PCEs.

- HPEG-PCE

HPEG-PCE is synthesized by using methyl allyl polyoxyethylene ether (HPEG) as the synthesizing macromonomer, which is a four-carbon monomer [27]. HPEG-PCE is predominantly synthesized via free-radical copolymerization. By systematically modulating the molar ratio of acrylic acid (AA) to HPEG, polymers with tunable anionic charge densities and variable side-chain lengths can be precisely engineered [28]. Regarding clay sensitivity, Schmid investigated the clay sensitivity of HPEG-PCE with different anion charge densities. The results demonstrated that the required dosage of highly anionic HPEG-PCE was 0.13% bwob to achieve a target flowability of 26 cm in pure calcined clay, significantly lower than the 0.3% bwob needed for its low charge density counterpart [20]. Due to its exceptional water-reducing performance and relatively low production costs, this material has become a dominant product in the Asian PCE market [29].

- TPEG-PCE

Isopentenyl polyoxyethylene ether (TPEG), a five-carbon macromonomer, serves as the foundational building block for synthesizing TPEG-PCEs. Owing to its mild polymerization conditions and superior performance relative to other ether-type PCEs, TPEG-PCE has emerged as the dominant commercial superplasticizer for slump retention in contemporary construction materials markets [30]. Maleic anhydride (MA) is a commonly used monomer when synthesizing TPEG-PCE. The incorporation of MA during synthesis enables the formation of shorter side chains and increased carboxylate group density, thereby significantly enhancing the fluidity of cement paste systems [31]. The limited reactivity of MA in polymerization reactions requires the incorporation of highly reactive co-monomers such as AA and methacrylic acid to achieve the desired performance characteristics [32]. Additionally, systematic optimization of TPEG-PCE post-addition temperature (the predetermined temperature regime required to be precisely controlled in the reaction system following initiator introduction) during synthesis has been shown to significantly improve slump retention capabilities [33].

- EPEG-PCE

Ethylene-glycol monovinyl polyethylene glycol (EPEG) (a new type of 2 + 2 monomer) is used as the synthesized macromonomer [34]. Conventional HPEG and TPEG monomers exhibit a low retention rate of unsaturated double bonds during polymerization, resulting in low molecular weight of PCEs [35]. In contrast, the unsaturated double bonds in EPEG exhibit significantly higher reactivity and require lower reaction temperatures, resulting in markedly improved polymerization efficiency [36]. In addition, EPEG-PCE also has performance characteristics such as strong adaptability, low sensitivity, and good slump preservation. At present, EPEG-PCE has been gradually commercialized and has excellent development prospects [37]. However, current studies on the clay sensitivity of EPEG-PCEs remain limited, making it challenging to establish a precise definition of EPEG-PCEs’ clay sensitivity.

The chemical structures of different types of PCEs are shown in Table 1 below.

### 2.2. Interaction Between PCE and Clay

#### 2.2.1. Competitive Adsorption Between Clay and Cement

**Figure 1:**
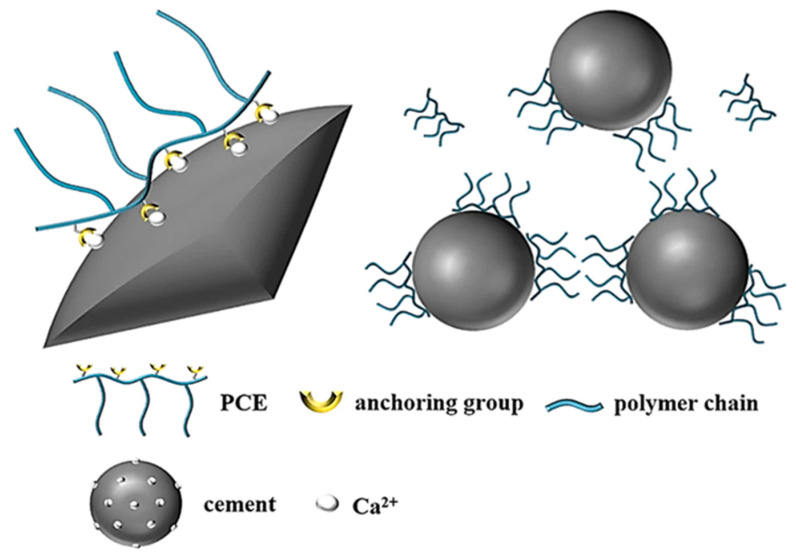
Schematic diagram of adsorption (**left**) and dispersion (**right**) of PCE [44].

PCEs are comb-shaped polymers composed of anionic main chains and PEO side chains [38]. The interactions between PCEs and cement particles manifest through five fundamental mechanisms: (1) steric hindrance effect, (2) electrostatic repulsion, (3) squeeze-out effect, (4) lubrication and wetting effect, and (5) air-entrainment and isolation effect [39,40,41]. This paper primarily examines the steric hindrance and electrostatic repulsion mechanisms. Cement typically contains mineral components such as tricalcium aluminate (C_3_A) and tetracalcium ferroaluminate (C_4_AF) [42]. During cement hydration, substantial Ca^2++^ ions are liberated, creating numerous cationic adsorption sites on cement surfaces. The anionic functional groups along the PCE backbone adsorb onto these sites through electrostatic interactions with surface Ca^2+^, thereby inducing electrostatic repulsion forces [38,43] (Figure 1).

**Figure 2:**
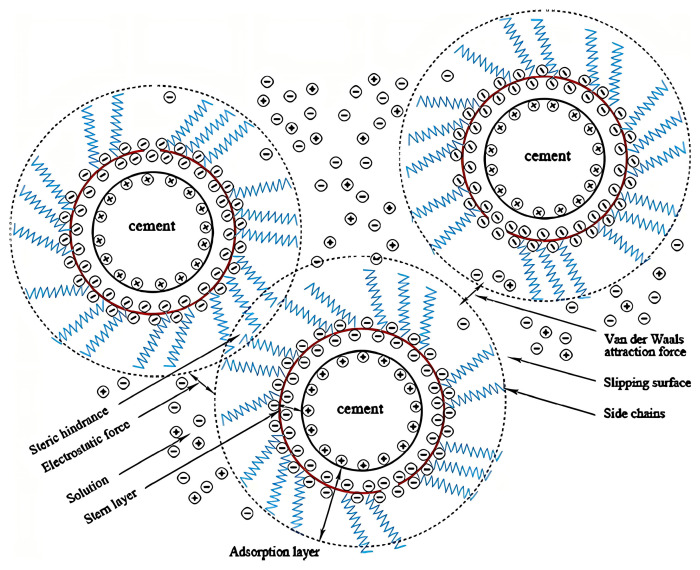
Schematic figure for adsorption and repulsion of cement particles with the PCEs [48].

The higher the concentration of PCE, the more negative charges on the surface of the cement particles, which in turn enhances the electrostatic repulsion [45]. This leads to the disintegration of cement particles’ flocculated structure, releasing the free water trapped within the flocculated structure, and thus improving the fluidity of the cement paste. Meanwhile, some scholars believe that steric hindrance plays a role. When the main chain of PCE which has negative electric charges adsorbs on the surface of cement particles, the PEO side chains will extend into the pore solution, forming a polymer adsorption layer on the surface of cement particles. When cement particles approach each other, the polymer adsorption layers will overlap (see Figure 2). Then, steric hindrance will be formed which promotes the dispersion of cement particles [40,46]. Studies have shown that the steric hindrance effect mainly depends on the length and density of the side chains. When the length of the side chain of the PCE is increased and the side-chain density is higher, the steric hindrance effect becomes more pronounced, which in turn enhances the fluidity of the cement [44,47].

However, with the rapid industrialization of the world, the production of concrete has soared globally [49,50]. As the demand for sand and gravel continues to rise, the availability of high-quality sand and gravel resources is diminishing, which contributes to the use of lower-quality sand in concrete construction. This shift has led to a higher clay content in concrete aggregates. Over time, it has become increasingly evident that clay has a significant detrimental impact on the performance of PCEs because of the competitive adsorption of PCE between clay and cement particles [11]. For example, Ng et al. [51] quantitatively demonstrated that montmorillonite (MMT) exhibits an exceptionally high adsorption capacity for PCEs, reaching 415 mg/g—approximately two orders of magnitude greater than that of ordinary Portland cement. This finding conclusively establishes that in clay-contaminated concrete systems, PCEs preferentially adsorb onto clay minerals rather than cement phases. So PCE does not play its due dispersion role [52] and the fluidity of cement slurry is greatly reduced. Because the particle size of clay minerals is very small (MMT particle size is between 0.5 and 10 μm), it results in a larger specific surface area of clay than the cement particles, making it easier for PCE to adhere to clay [53].

**Figure 3:**
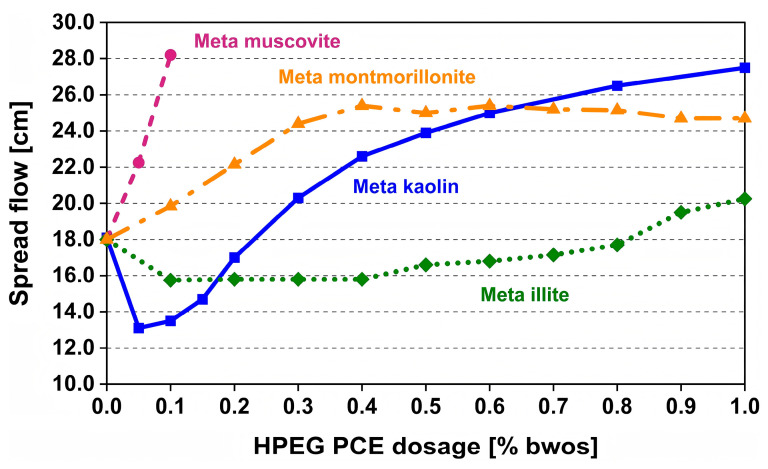
Dosage-dependent dispersing behavior of PCE sample in pastes prepared from the meta clay samples suspended in synthetic cement pore solution [56].

In order to investigate the competitive adsorption mechanism, there are more and more scholars who have studied the interaction between other clay minerals and PCEs. Zhang et al. systematically investigated the interactions between PCEs and kaolinite particles in alkaline aqueous suspensions (pH = 8.3) [54]. They found that at low PCE concentrations (≤0.6 wt.%), the viscosity of the suspensions increased significantly, exceeding 100 times the original value. However, when the PCE concentration was further increased (≥3 wt.%), the viscosity returned to its original level. This behavior indicates that kaolinite can interact with PCE through surface adsorption. Muscovite can also significantly affect the dispersion ability of PCE superplasticizers, leading to a decrease in the fluidity of cement paste. Chi et al. added 6 wt% muscovite to the cement paste [55]. At a PCE dosage of 0.14%, the spread flow value decreased by 51%. In the cement paste mixed with muscovite, the ion concentration in the pore solution increases due to the dissolution of muscovite, thereby reducing the critical micelle concentration of PCE in the pore solution, promoting the aggregation of PCE, and resulting in a decrease in its surface activity and adsorption energy. Schmid and her colleagues investigated the interactions between various clay minerals and PCEs [56]. By setting the initial spread flow value to 18 cm, they experimentally determined the spread flow values of kaolinite, muscovite, MMT, and illite at different PCE dosages. The results indicated that kaolinite, muscovite, and MMT all responded positively to PCE addition. Specifically, when the PCE dosage reached 0.2% bwoc, the spreading flow of cement containing muscovite exceeded 28 cm. Meanwhile, the maximum spread flow for cement containing kaolinite and MMT was 24 cm, achieved at a PCE dosage of 1% bwos. However, the spread flow value of cement containing illite showed minimal improvement (Figure 3). Xiong et al. also explored the adsorption capacity of PCE on different clays and found that the sequence of the adsorption capacity was MMT (almost 90%) > kaolinite > illite [57].

The preceding analysis demonstrates that clay minerals influence PCE performance differentially, with variability principally arising from their distinct crystal structures and lattice charge characteristics, as detailed in the following discussion.

#### 2.2.2. Mechanism of Interaction Between PCE and Clay

Clay minerals are natural materials composed of fine-grained minerals which are less than 2 microns in size. They are layered structured silicates composed of SiO_4_ tetrahedrons and AlO_6_ octahedrons [58]. Clay minerals are fundamentally classified as 1:1 or 2:1 phyllosilicates according to their layered crystal structures [59]. Representative clay minerals include kaolinite, muscovite, illite, and MMT.

**Figure 4:**
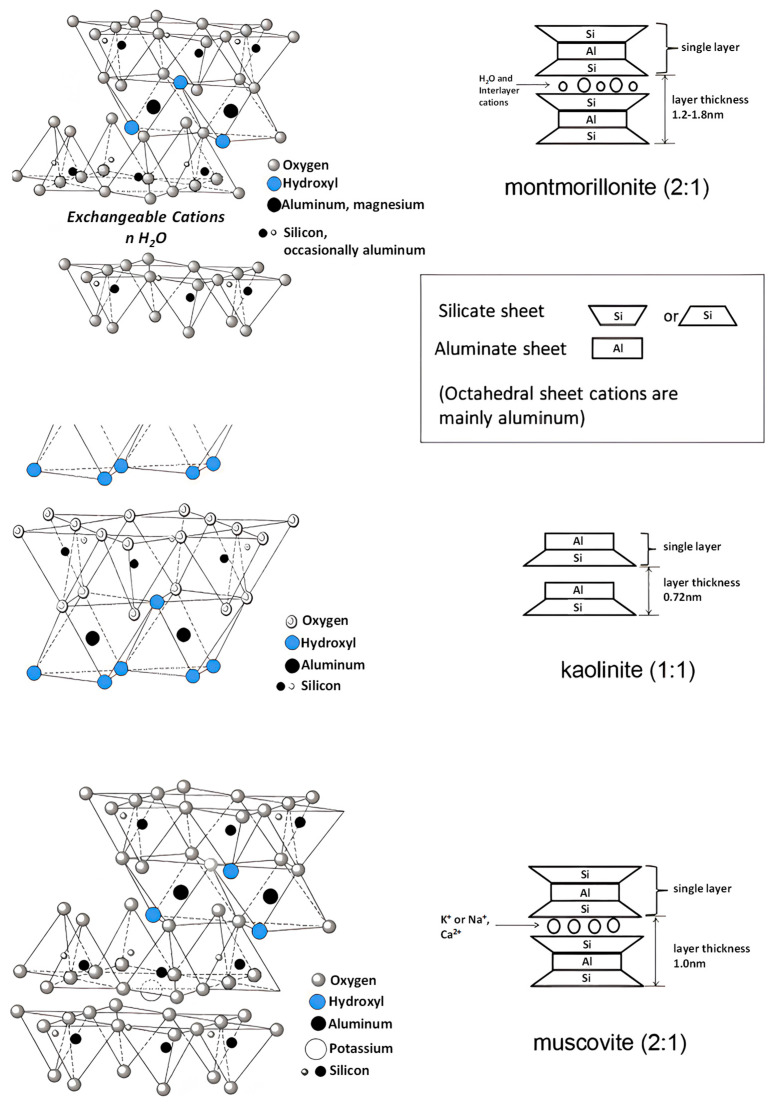
Structural diagram of different types of clay [62].

Kaolinite (chemical formula Si_2_Al_2_O_5_(OH)_4_) is a 1:1 layer silicate mineral (see Figure 4). It is composed of a 1:1 alternating stack of SiO_4_ tetrahedral sheets and AlO_6_ octahedral sheets [60]. The structural integrity of 1:1 phyllosilicates arises from hydrogen bonding between hydroxyl groups (OH^−^) in AlO_6_ octahedral sheets and oxygen atoms in adjacent SiO_4_ tetrahedral sheets, with an absence of interlayer cations. Muscovite (KAl_2_(Si_3_AlO_10_)(OH)_2_) and illite (chemical formula Si_4_(Al,Mg,Fe)_2.3_O_10_(OH)_2_ (K,H_2_O)) (Figure 4) are non-expanding 2:1 layer silicates, where the AlO_6_ octahedral sheets are sandwiched between two SiO_4_ tetrahedral sheets that are linked by electrostatic force [61]. These minerals contain limited interlayer cations. In addition, MMT (chemical formula (Na,Ca)_0.3_(Al,Mg)_2_Si_4_O_10_(OH)_2_·nH_2_O) is also a 2:1 layer silicate (Figure 4). Unlike muscovite and illite, MMT has fewer interlayer charges (0.2–0.5 interlayer charges per 11 oxygens, compared to 0.5–0.75 in illites) [62]. So the layers in MMT are held together primarily by van der Waals forces. The weak interlayer bonding forces are readily disrupted by water molecule intercalation, resulting in increased layer spacing and pronounced swelling behavior. The presence of Na^+^ or Ca^2+^ ions results from the substitution of Mg^2+^, Fe^2+^, or Al^3+^ in the octahedral sheets, leading to a charged layer, and water molecules occupy the interlayer space between the octahedral sheets [63].

**Figure 5:**
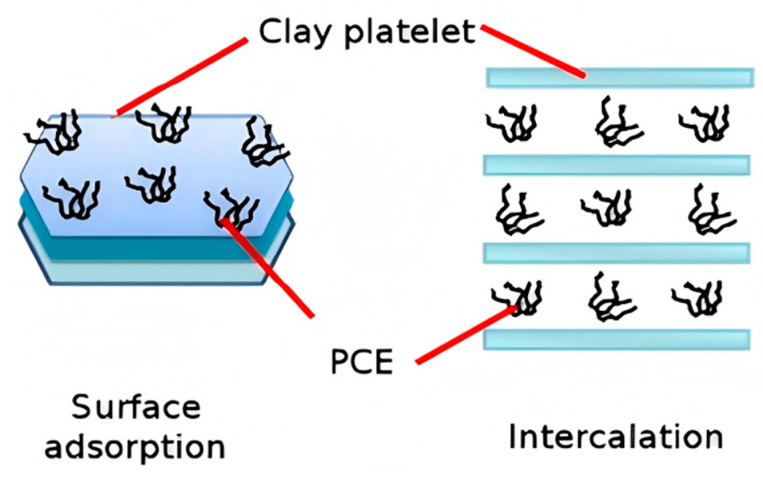
Schematic figure for interaction between clay and PCE [70].

The distinct interaction mechanisms between PCEs and various clay minerals are systematically summarized in Table 2, with comprehensive analysis provided in the following discussion. To summarize, the effects of clay minerals on PCE are mainly surface adsorption and chemical intercalation [64,65] (Figure 5). Clay mineral surfaces and edges exhibit negative surface charges, preventing direct adsorption of PCEs due to electrostatic repulsion. However, upon cement hydration, the pore solution becomes enriched with divalent Ca^2+^ cations, which readily adsorb onto the negatively charged clay surfaces to form a cationic interfacial layer. Li et al. confirmed the adsorption of cations such as Ca^2+^ on the surface of MMT through the Zeta potential test [66]. The anion groups in the PCE can chelate with the metal cation adsorption layer, making PCE further adsorbed on the clay [67]. Muscovite and illite are three-layer structures, so there are more negative charges on their surfaces to obtain electrostatic adsorption. Furthermore, previous studies have confirmed that PCE can also adsorb onto kaolinite surfaces through electrostatic interactions [68,69].

**Figure 6:**
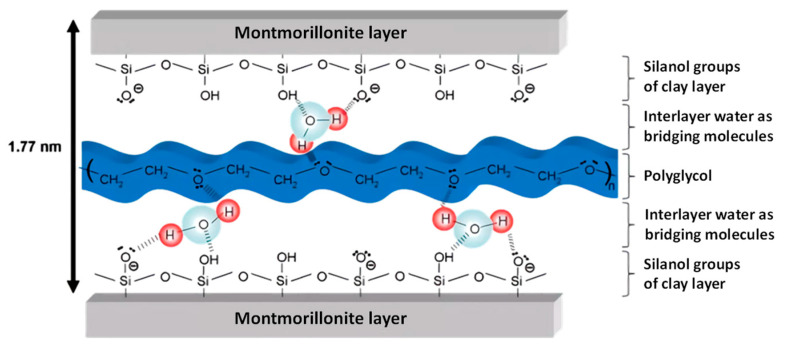
Schematic figure for PCE intercalates in the MMT interlayer [51].

Another interaction mechanism involves chemical intercalation, which is particularly relevant for MMT due to its weak interlayer bonding and expandable 2:1 layered structure. When the water is added to the concrete, the H_2_O molecules will first enter the MMT interlayer, and the distance between the MMT layers will increase due to the entry of H_2_O molecules. Then, the polymer will enter the MMT interlayer. At this time, the O in the ether bond on the PCE side chain and the O on the MMT will bridge the H in the water molecules to form a hydrogen bond [71], and further intercalate in the MMT interlayer (Figure 6). Ma et al. conducted XRD and adsorption tests for MMT with different concentrations of PCE [72]. They concluded that when the added amount of PCE was at a low concentration, it was mainly adsorbed on the surface of MMT. When the concentration of PCE increased, PCE was mainly adsorbed by inserting side chains into the interlayer of MMT.

## 3. Methods of Improving PCE’s Clay Sensitivity

### 3.1. Side Chain

#### 3.1.1. Reduce the Length and Density of Side Chain

**Figure 7:**
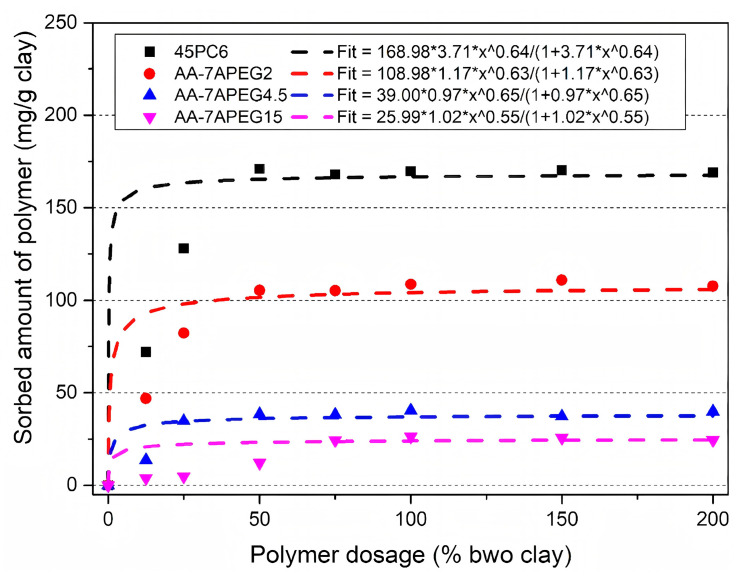
Sorption isotherms for various polymer samples with sodium bentonite dispersed in synthetic cement pore solution (pH = 13, solution/clay ratio = 48; the term A*B represents the multiplication between A and B) [26].

The side chain of PCE is easily inserted into the clay interlayer, resulting in PCE loss. The elongation of PEO side chains in PCEs correlates with an increased density of ether functional groups. So it is easier to form hydrogen bonds with the active sites in the clay interlayer and adsorb in the clay interlayer [73]. Consequently, reducing the PEO side-chain length in PCEs effectively suppresses intercalation adsorption onto clay minerals [21]. Yang et al. explored the adsorption behavior of PCE on MMT [74]. With a long side chain, the adsorption capacity of PCE on MMT could reach 30 mg/g, while the maximum adsorption capacity of a short side-chain structure was 9 mg/g. Similarly, Lei et al. prepared a series of APEG-PCEs with short side-chain lengths (n_EO_ = 7) [26]. Under fixed PCE dosage (0.15% bwoc), the addition of 3% bwoc clay reduced the slump flow of cement containing long side-chain PCEs by 73%, whereas the slump flow of cement containing shorter side-chain PCEs only decreased 40% under identical conditions. Clay adsorption experiments also show that when the side-chain density is low (higher acid/ether ratio), PCE has a lower side-chain size, which reduces the intercalation of PCE and enables the PEO side chain to play a dispersive role (Figure 7). Although reducing the length and density of PEO side chains in PCEs mitigates clay sensitivity, this modification concurrently decreases steric hindrance effects essential for effective dispersion of cement particles. Therefore, further research is needed to optimize this approach.

#### 3.1.2. Introduce Large-Volume Groups

**Figure 8:**
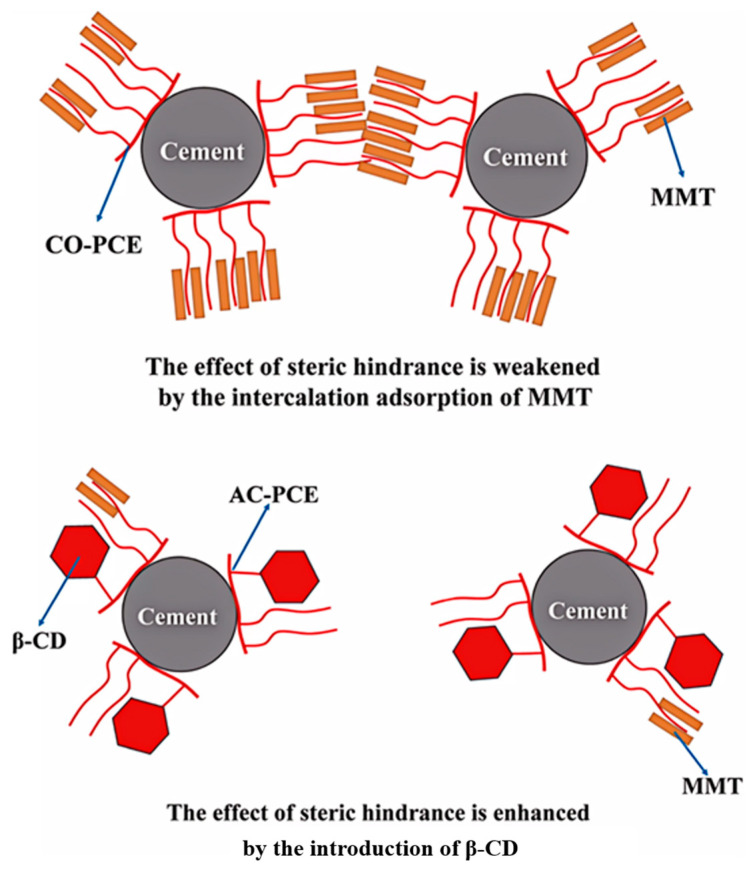
Schematic figure for inhibiting clay sensitivity by grafting β-CD [76].

The steric hindrance of PCE can be effectively enhanced through strategic incorporation of bulky substituents into its side-chain structure. By design, the bulky substituents of PCE exceed the clay interlayer spacing, sterically blocking PEO intercalation and thereby enhancing clay resistance [69]. Yao et al. synthesized a PCE with a large polysaccharide structure side chain by introducing modified sodium alginate [75]. At 1.5% MMT content, conventional comb-type PCE yields a cement slurry fluidity of merely 18 cm, while the cement slurry containing modified sodium alginate PCE demonstrates significantly improved flowability (24 cm). Due to the steric hindrance caused by its large conformation size, the PEO side chain was prevented from being inserted into the MMT interlayer. Similarly, Li et al. grafted β-cyclodextrin (β-CD) in the traditional PCE side chain [76] (as shown in Figure 8). The three-dimensional β-CD structure (maximum diameter: 1.53 nm) exceeds the MMT interlayer spacing, preventing intercalation and thereby enhancing PCE’s steric resistance to mitigate clay sensitivity. These results demonstrate that incorporating sterically hindered groups (e.g., polysaccharides or β-CD) into PCE side chains effectively mitigates clay sensitivity. This approach simultaneously enhances PCE dispersion in clay-contaminated cement systems and provides a viable pathway for developing high-performance, clay-tolerant PCEs.

### 3.2. Main Chain

#### 3.2.1. Introduce Anionic Groups

**Figure 9:**
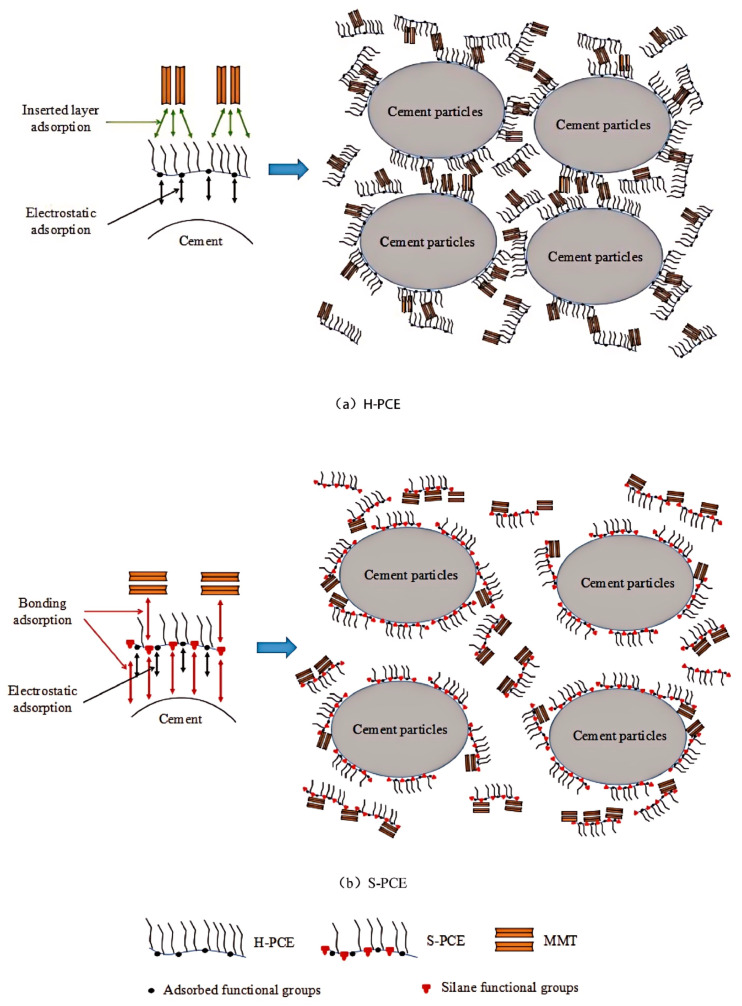
Schematic diagram of dispersion and adsorption mechanism of H-PCE and S-PCE [78].

Anionic group incorporation has been widely employed as an alternative approach to reduce PCE’s clay sensitivity. The investigation centers on two key anionic groups. The first is the anionic group containing the sulfonic acid group. Guo et al. introduced 2-acrylamyl-2-methylpropanesulfonic acid (AMPS) into the PCE side chain [77]. Systematic increase in the AMPSA/HPEG molar ratio (0.1:1 baseline) improved MMT-contaminated paste flowability by 22% (from 135 mm to 165 mm). This enhancement originates from the sulfonate groups in AMPS, which preferentially adsorb onto MMT surfaces, generating electrostatic repulsion that simultaneously prevents PCE intercalation into the clay interlayers. So this way is conducive to inhibiting the clay sensitivity. Wang et al. developed an anti-clay S-PCE by incorporating γ-methacryloyloxypropyltrimethoxysilane (KH570) [78]. XRD analysis revealed that the MMT interlayer spacing expanded by 0.53 nm in the HPEG-PCE solution but only 0.03 nm in the S-PCE solution, demonstrating significantly suppressed intercalation. The silanol groups (Si-OH) in S-PCE dehydrate and bond to the silanol on the surface of the MMT, promoting surface adsorption over interlayer insertion (Figure 9). This mechanism effectively blocks PEO side-chain intercalation, preventing clay-induced performance degradation [79,80]. Furthermore, Li et al. improved both main-chain rigidity and PCE spatial extension through the incorporation of the clay-resistant sodium methylpropylene sulfonate group, while optimizing the raw material ratios [81]. The synthesized PCE has good workability with cement mortar containing MMT.

**Figure 10:**
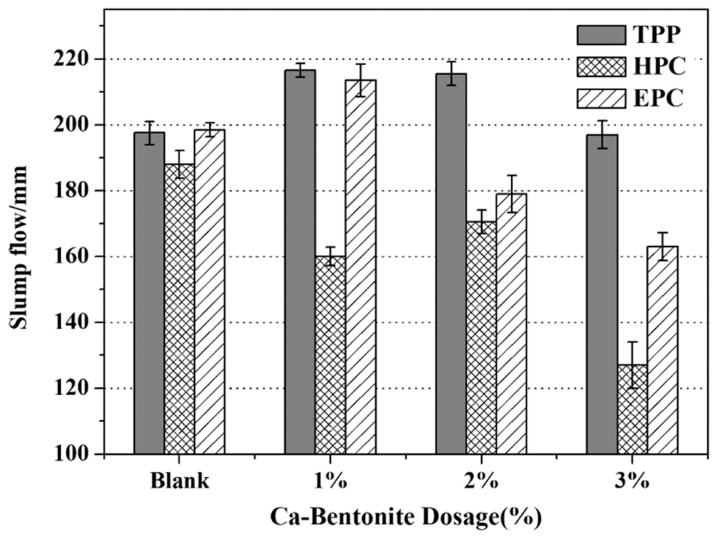
Effect of PCE type on the initial fluidity of mortar with different dosage of Ca-bentonite [83].

In addition to sulfonic acid groups, Zhang et al. designed a phosphonate-modified PCE (PHS-PCE) [82]. In clay-containing systems, PHS-PCE demonstrated superior adsorption stability with only an 18% reduction in cement adsorption capacity, while conventional PCE showed a 25% reduction. The phosphonate groups in PHS-PCE preferentially adsorb onto clay particle edges, effectively blocking PCE intercalation and thereby mitigating the clay sensitivity of PCE. Similarly, Feng et al. introduced phosphate groups as side chains into TPEG-PCE to prepare a clay-tolerant PCE (TPP) [83]. When containing 1% clay, TPP yielded mortar fluidity of 218 mm, substantially exceeding HPEG-PCE’s performance (160 mm) at equivalent dosages (Figure 10). Only when the dosage of HPEG-PCE was increased by approximately 20% did the initial fluidity of the mortar approach that of TPP, demonstrating TPP’s superior clay tolerance and dispersion stability.

Functionalizing PCE with anionic groups, particularly sulfonic acid and phosphonate moieties, effectively addresses clay sensitivity through suppressed interlayer adsorption, thereby preserving PCE’s intended performance characteristics. The studies by scholars above collectively demonstrate that tailored PCE modifications can significantly diminish clay sensitivity, offering practical solutions for improving the performance of cementitious materials in clay-contaminated environments. It is critical to meticulously regulate the proportion of the introduced functional groups relative to other raw materials. An excessive molar ratio of these groups may yield counterproductive effects, potentially aggravating rather than alleviating clay sensitivity.

#### 3.2.2. Amphoteric Polycarboxylate Superplasticizer

**Figure 11:**
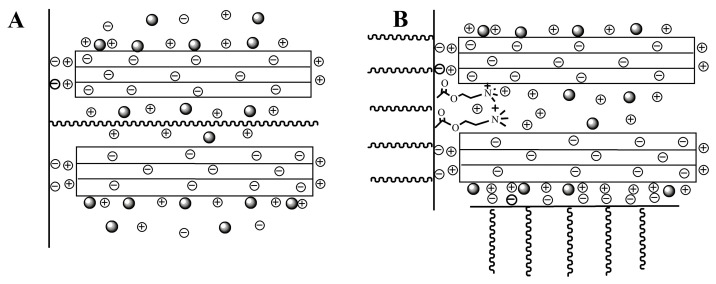
Interaction mechanism between APC and sodium bentonite. ((**A**)—for PCE and (**B**)—for APC) [86].

Amphoteric polycarboxylate superplasticizers (APCs), incorporating both anionic and cationic functional groups, represent specialized PCEs. Their cationic moieties predominantly comprise quaternary ammonium and primary amine groups [84,85]. These APCs can exhibit cation exchange intercalation effects with clay minerals, allowing the cationic groups to insert into the interlayer spaces of clay. This intercalation competes with hydrogen-bonding interactions, thereby inhibiting the penetration of PEO side chains into the interlayer and mitigating the detrimental effects of clay on the fluidity of cement slurries. Tang prepared three APCs by introducing three cationic monomers, such as methylacryloxyethyl trimethyl ammonium chloride (DMC) and acrylamide [86]. The quaternary ammonium cation in DMC undergoes ion exchange with the clay interlayer, further promoting intercalation, thereby preventing the penetration of PEO side chains into the clay layers (Figure 11).

**Figure 12:**
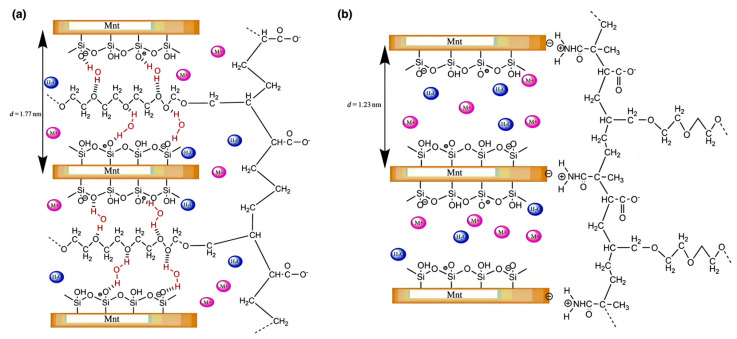
Schematic diagram of MMT with PCEs: (**a**) c-PCE and (**b**) ct-PCE [88].

Compared to conventional PCEs without cationic monomers, these APCs demonstrated enhanced adsorption on cement particles (96.58 mg/g) and improved fluidity (90 mm higher than that of ordinary PCE) in cement slurries containing 1% bentonite. Similar findings were reported by Zhang et al. through analogous experiments [87]. Furthermore, Ren et al. developed a ct-PCE with amide cations and shorter side chains [88]. The adsorption amount of commercial PCE with PEO side chains can reach up to 200 mg/g, while only a small amount of ct-PCE (<25 mg/g) is adsorbed by Na-MMT, which significantly demonstrates that ct-PCE exhibits reduced clay sensitivity. Because the MMT surface and edge are negatively charged, the amino cations in PCE can be adsorbed on the MMT surface by electrostatic adsorption rather than being inserted into the interlayer (Figure 12), which is different from the action mechanism of quaternary ammonium ions. Therefore, APCs can inhibit clay sensitivity by two distinct mechanisms: (1) quaternary ammonium cations participate in ion exchange with interlayer cations, while (2) amino groups undergo electrostatic surface adsorption. However, these findings contrast with the conclusions drawn by some other scholars [89,90]. Thus, it is necessary to investigate the mechanism of interaction between APCs and clay in the future, so that a more precise and widespread application of this approach can be achieved.

### 3.3. Change Traditional Comb Structure

**Figure 13:**
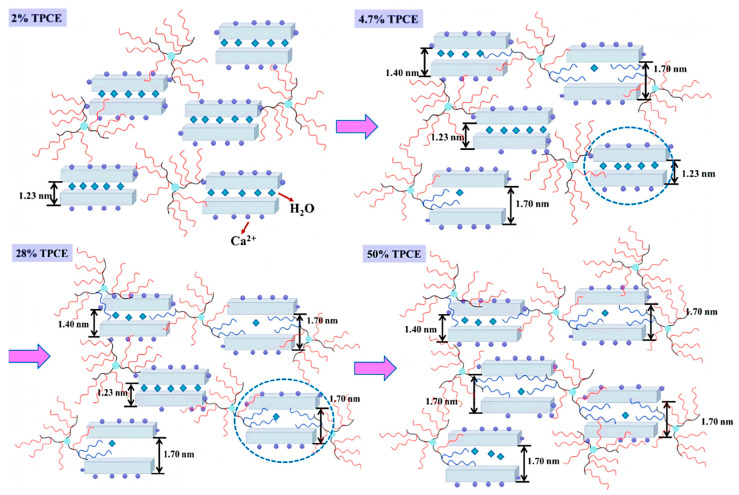
Intercalation modes of the synthesized PCEs on MMT [93].

In addition to comb-shaped structures, an increasing number of new topological structures of PCE have been synthesized, such as cross-linked structures, star-shaped structures, claw-shaped structures, and hyperbranched structures [40,91,92]. By modifying the topological structure of PCEs, the steric hindrance effect of PCE can be enhanced, and the adsorption and intercalation of PCE on clay can be reduced. Employing trimethylolpropane triacrylate as a cross-linker, Ma et al. synthesized a cross-linked structure PCE (TPCE) with TPEG and AA [93]. At 0.5% MMT content, CPCE and TPCE slurry fluidities measured 266.5 mm and 281.0 mm, respectively. When MMT increased to 3%, CPCE slurry exhibited an *R_f_* value of 68.1%, while TPCE maintained 153.73 mm fluidity with an *R_f_* value of 45.9% (*R_f_* refers to the relative flowability change, which can be calculated by Equation (1)). Different from traditional comb-like PCE, a single TPCE can intercalate multiple MMT particles (Figure 13). Its multi-branch structure reduces the number of intercalated molecules and the loss of TPCE, thereby endowing it with strong adsorption, dispersion, and dispersion retention capabilities for cement containing clay.

(1) $$R_{f}=\frac{f_{c0}-f_{cw}}{f_{c0}}\times 100$$

where *f*_*c*0_ and *f_cw_* are the flowability of pastes containing 0% and w% MMT, respectively.

**Figure 14:**
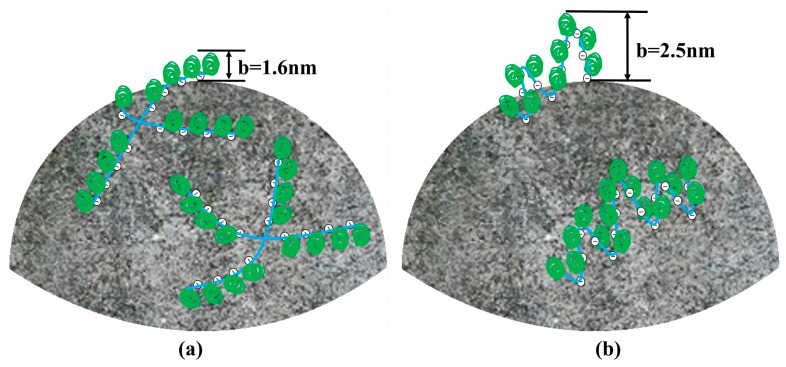
Schematic representation of the actions of SPCE and CPCE on cement surfaces; (**a**) for SPCE; (**b**) for CPCE [95].

Liu et al. synthesized a star-shaped structure PCE by inducing a star-shaped structure core [94]. When 0.15% bwoc of PCE is added, the fluidity of the cement slurry containing SPCE exceeds 325 mm, which is approximately 50 mm higher than that of ordinary comb-shaped PCE. By changing the PCE topological structure from comb-like to star-shaped, it was endowed with a strong steric hindrance to improve slurry flow and flow retention. Similarly, Lai et al. synthesized a star-shaped four-arm PCE through atom transfer radical polymerization [95]. Star-shaped PCE adsorbs onto the cement surface in a flatter conformation, whereas conventional PCE adsorbs in a more curved conformation (Figure 14). This difference results in the steric hindrance caused by star-shaped PCE being 89% greater than that of conventional PCE. The new topological structure greatly improved the dispersion performance and flow retention performance. Although the change in topological structure provides a certain possibility to suppress the sensitivity of clay by stronger steric hindrance, it makes the PCE synthesis process more complicated such as the introduction of cross-linking agents, which inevitably increases the production cost. Thus, streamlined and economical synthesis routes remain critically needed.

Through the above analysis, several strategies have been explored to mitigate the negative effects of clay on the flow and flow retention performance of PCE based on the structure of PCE. These strategies include (1) controlling the length and density of the side chains, (2) introducing groups that inhibit clay sensitivity, and (3) modifying the topological structure. The detailed elaboration of these methods is presented above, with a systematic summary provided in Table 3.

### 3.4. Add Anti-Clay Sacrificial Agent

Beyond molecular-structure optimization of PCEs, sacrificial clay inhibitors have emerged as a cost-effective and operationally simple alternative for mitigating PCEs’ clay sensitivity [16,96]. The characteristics of various sacrificial agents are elaborated in detail below and systematically summarized in Table 4.

#### 3.4.1. Ionic Sacrificial Agent

Cationic sacrificial agents are typically synthesized using monomers such as dimethyl diallyl ammonium chloride, which electrostatically adsorb onto negatively charged clay surfaces [105,106]. For instance, Wang et al. introduced dimethylamine cation groups and acrylamide to synthesize a terpolymer as a cationic sacrificial agent [97]. When incorporating 0.20% sacrificial agent with PCE, the MMT-containing cement slurry maintained a fluidity of 270 mm after 120 min. By contrast, the reference system without a sacrificial agent showed substantial flow loss, with fluidity decreasing to only 140 mm under identical conditions. This finding demonstrates that cationic sacrificial agents can mitigate the adverse effects of MMT on cement slurry performance by occupying the active sites on the clay surface.

**Figure 15:**
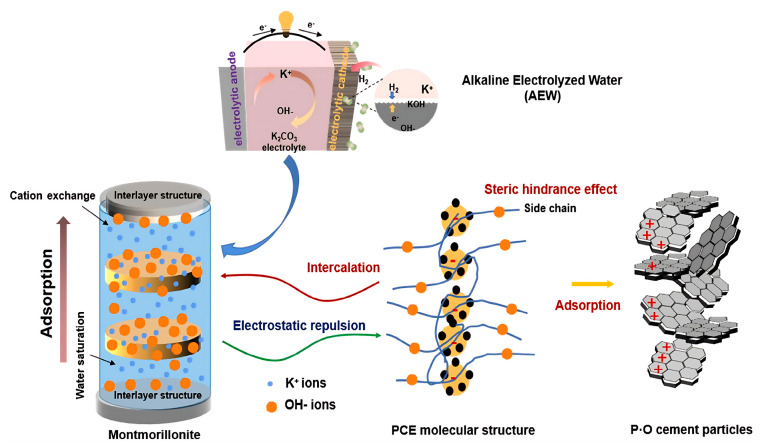
Mechanism analysis of clay-resistance for AEW-based mortar containing clay [99].

Gómez et al. demonstrated a positive correlation between molecular weight and clay surface adsorption capacity for quaternary amine sacrificial agents [98]. This adsorption significantly reduces the specific surface area of the clay. Similarly, Li et al. demonstrated that using alkaline electrolyte water (AEW) instead of ordinary tap water during cement hydration can increase the surface charge density of cement particles [99]. MMT exhibits an 89% adsorption rate to PCE superplasticizer in tap water within one hour, decreasing to 72% in AEW. The highly active metal cations in AEW adsorb onto the surface of cement, enhancing the adsorption of PCE. In the presence of clay, these cations preferentially intercalate into the interlayer structure of the clay until reaching saturation (Figure 15). This process alleviates the chemical intercalation effect of PCE side chains, thereby achieving an anti-clay effect to prevent clay-induced fluidity loss.

The aforementioned methods effectively mitigate the interaction between PCE and clay through cation adsorption on the clay surface or within the interlayer, as validated by experimental results. However, it is essential to consider the associated economic implications. For instance, the cement hydration process demands a substantial volume of water. Replacing tap water with alkaline electrolyzed water would substantially increase production costs, thereby limiting the practical applicability of this approach.

#### 3.4.2. Non-Ionic Sacrificial Agent

In addition to ionic sacrificial agents, non-ionic sacrificial agents are also employed to mitigate clay sensitivity. For instance, Li et al. developed a polyethylene glycol (PEG)-grafted lignin as an anti-clay sacrificial agent [100]. When 0.012 wt% PEG-grafted lignin was added to MMT-containing cement slurry (*w*/*c* = 0.29), the fluidity of the slurry was 240 mm, 40 mm larger than that of the blank slurry. The strong steric hindrance of lignin effectively prevented the adsorption of PCE and other molecules, while the insertion of PEG into the interlayer of MMT hindered the intercalation of PCE. That can be proved by the D-spacing of MMT moving from 1.23 nm to 1.52 nm when PEG-grafted lignin was mixed with MMT. He et al. synthesized non-ionic sacrificial agents based on various polyols [101]. Their results revealed an inverse correlation between hydroxyl group density in polyols and MMT adsorption energy. The adsorption energy in xylitol is −6.75 eV, whereas the highest adsorption energy of −2.70 eV is observed in diethylene glycol (a diol). The higher the number of hydroxyl groups in the polyols leads to enhanced MMT adsorption capacity. Additionally, the combination of these polyols with PCE enhanced the tolerance to clay minerals. There are few studies on non-ionic sacrificial agents, but more preparation of such sacrificial agents can be explored in the future based on the mechanism of surface adsorption and steric hindrance.

#### 3.4.3. Swelling Inhibitor

**Figure 16:**
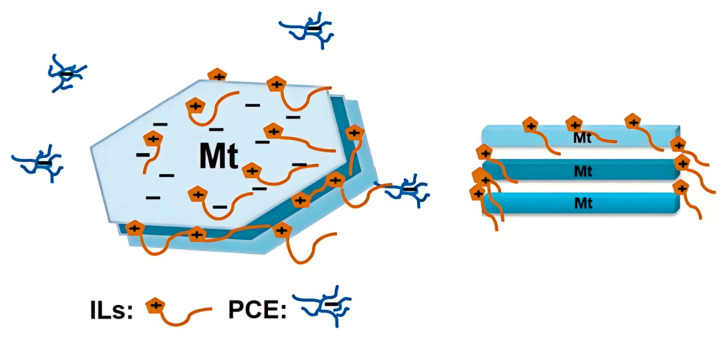
Mechanism of the improvement of ILs in MMT tolerance of PC [102].

Due to the high swelling rate and hydrophilicity of MMT, the water reduction efficiency of PCE is seriously hindered. Therefore, the swelling rate of MMT can be reduced and hydration can be prevented by adding clay swelling inhibitor, so as to isolate the interaction between PCE and MMT [107,108]. Ionic liquids (ILs) are organic molten salts composed of organic cations and inorganic/organic anions. He and coworkers synthesized a series of ionic liquids as sacrificial agents containing different long-chain imidazolium-based cation and anion to apply to cement paste with MMT [102]. As shown in Figure 16, ILs have a stronger ability to interact with MMT and can be preferentially absorbed by MMT compared with PCE. The addition of ILs composed of different long-chain imidazolium-based cation and different anions could increase tolerance for clay of PCE, making PCE more compatible in cement paste containing clay, improving the fluidity of cement–MMT paste significantly by 70%.

**Figure 17:**
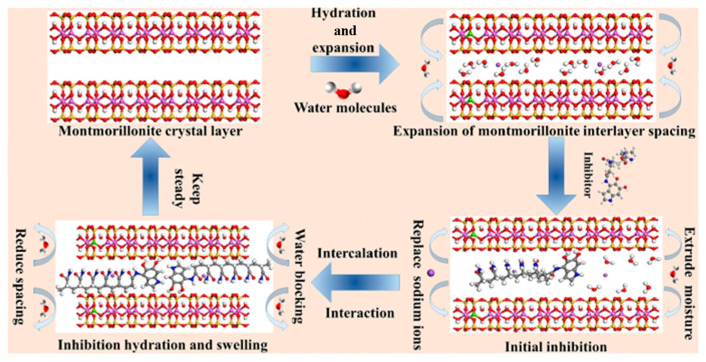
The behavior of P(AM-DA) molecules in clay crystals layer [103].

Guan et al. synthesized a dopamine-derivative-grafted polymer P(AM-DA) [103], which can be used to resist the negative effects of clay. The groups on the P(AM-DA) such as the catechol groups and protonated amine groups allowed the polymer to stick tightly to the clay surface. Protonated amine groups diminish interlayer electrostatic repulsion, enabling the formation of a uniform surface layer that restricts water molecule permeation (Figure 17). It can be proven by the Zeta potential of the clay particles significantly changing from −41.2 mV to −0.33 mV after 1 adding P(AM-DA). In addition, Khandelwal et al. used a silane coupling agent to modify MMT [104]. Due to the cation exchange between the silane coupling agent and the alkali metal ions in the MMT layers, the intercalation of PCE into the MMT interlayer was reduced, thereby enabling PCE to maintain the fluidity of the cement slurry. Silane modification enhanced hydrophilicity, increasing mortar flow from 26.89% to 65.25%. Additionally, the exchange of interlayer alkali cations by silane reduced the swell index by 84%, effectively controlling the swelling behavior of the clay.

Collective evidence demonstrates that clay swelling inhibitors selectively engage with clay minerals through active functional moieties, such as imidazolyl cations and amine groups. Two distinct inhibition mechanisms emerge: (1) surface adsorption forms a protective coating that restricts clay hydration and expansion, and (2) intercalation into clay interlayer occupies reactive sites, blocking water ingress and stabilizing interlayer spacing. Future research in this field holds considerable promise and merits further in-depth investigation.

## 4. Future Prospect

With the widespread adoption of PCEs in concrete formulations, significant advances have been made in elucidating the fundamental interaction mechanisms between PCE superplasticizers and clay minerals. Concurrently, three primary strategies are employed to mitigate the detrimental effects of clay on PCE performance in cementitious systems. The first approach employs a two-stage PCE addition strategy. A portion of PCE adsorbs onto the clay surface while the remainder intercalates into the interlayer spacing of clay aluminosilicate layers, functioning as a ‘sacrificial agent’ to mitigate the detrimental effects of clay. Subsequently administered PCE molecules preferentially adsorb onto cement particles, effectively dispersing the cement matrix and thereby enhancing the overall performance of the concrete system. The second method is using sacrificial clay inhibitors (e.g., lignosulfonate or polyethylene glycol) in a pretreatment step for contaminated sand, thereby preventing clay from compromising PCE effectiveness in the concrete system. The third strategy focuses on molecular structure optimization of PCEs to suppress their clay sensitivity, which is the most direct and fundamental way.

While these strategies have partially mitigated the detrimental impact of clay minerals on the water-reducing efficiency of PCEs, several critical limitations remain unresolved. For instance, although PCEs with short side chains exhibit reduced affinity for clay surfaces, their diminished steric hindrance—compared to long side-chain analogues—results in inferior cement particle dispersion. Furthermore, while the incorporation of bulky functional groups can enhance steric stabilization, this approach introduces synthetic complexities and escalates production costs. Additionally, the use of sacrificial agents requires meticulous optimization of dosage levels and thorough evaluation of their potential interactions with other concrete constituents.

Moving forward, future research should prioritize the development of simplified synthesis methods for anti-clay PCE while maintaining or enhancing its clay resistance. Additionally, exploring more cost-effective and efficient strategies will be critical to achieving high performance and facilitating the widespread application of PCEs in concrete. In addition, conventional PCEs typically retard the initial setting time of concrete, and structural modifications can further alter this effect. Consequently, future research on PCEs should not only focus on enhancing their clay resistance through molecular design but also systematically evaluate how such structural changes influence setting time (initial setting time and final setting time), ensuring balanced performance in both workability and hardening properties. Furthermore, it is worth noting that research on supplementary cementitious materials (SCMs; e.g., silica fume, slag, fly ash) as cement alternatives has advanced considerably. While this review focuses specifically on PCE interactions with cement and clay, the adsorption behavior and compatibility mechanisms between PCEs and SCMs may differ substantially. These differences warrant systematic investigation in future studies.

## 5. Conclusions

This paper reviews the research on the mechanism of clay sensitivity and the corresponding improvement of inhibiting PCEs’ clay sensitivity. The key findings are summarized as follows:

- Clay minerals exhibit competitive adsorption behavior with cement particles for PCEs through both physical adsorption and chemical intercalation mechanisms. Among common clay minerals, MMT demonstrates particularly detrimental effects on PCE performance due to its pronounced swelling characteristics and water absorption capacity, which originate from weak interlayer bonding forces and consequent interlayer expansion.
- By altering the molecular structure of PCE (adjusting the length and density of side chains, introducing bulky molecular groups, modifying the comb-shaped structure, introducing anion groups and cationic groups), the intercalation adsorption of PEO side chains can be minimized, thereby imparting clay resistance to PCE.
- Sacrificial agents primarily act by occupying the active sites on clay surfaces through ionic electrostatic adsorption or non-ionic polymer physical adsorption. Their presence prevents direct contact and reaction between clay and PCE.
- The swelling inhibitors mitigate the expansion and hydration of clay minerals by encapsulating the clay surface or intercalating into the clay interlayers. This reduces the expansion rate of clay and enhances its hydrophobicity, thereby diminishing the adverse effects of clay on PCE performance.

## Figures and Tables

**Table 1:** Chemical structure of different types of PCEs.

Category	Chemical Structure	Refs
MPEG-PCE	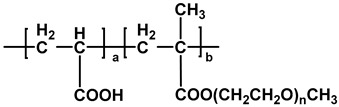	[21]
APEG-PCE	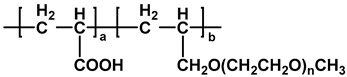	[26]
HPEG-PCE	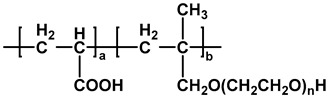	[28]
TPEG-PCE	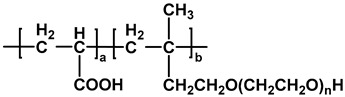	[32]
EPEG-PCE	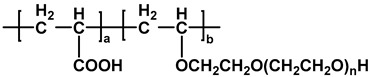	[35]

**Table 2:** Interaction mechanism between different clays and PCE.

Clay Minerals	Structure (SiO_4_:AlO_6_)	Interlayer Force	Mechanism	Refs
Kaolinite	1:1	Electrostatic force	Surface adsorption	[58,60]
Muscovite and illite	2:1	Electrostatic force	Electrostatic adsorption	[61,62]
Montmorillonite		van der Waals force	Intercalation adsorption	[51,63]

**Table 3:** Mechanism of reducing clay sensitivity based on molecular structure.

Molecular Structure	Factor	Functional Group/Structure	Mechanism		Refs
Side chain	Length	Ethoxy unit	Hydrogen bonding	Shortening the PCE side chains reduces hydrogen-bond formation with clay interlayers.	[21,74]
	Density	Carboxyl group	Charge density	A higher acid-to-ether ratio decreases side-chain density while increasing main-chain charge density, thereby enhancing surface adsorption.	[26]
	Bulky groups	Sodium alginate β-cyclodextrin	Steric hindrance	Bulky groups in side-chain enhance PCE steric hindrance, effectively preventing intercalation into clay interlayers.	[69,75,76]
Main chain	Anionic group	Sulfonic acid group	Electrostatic attraction	Anion groups can act as anchor points and strengthen the adsorption on the clay surface through electrostatic attraction, which reduces intercalation.	[78,79,81,83]
		Phosphate groups			
	Cationic group	Quaternary ammonium	Cation exchange	Quaternary ammonium cations undergo ion exchange with clay interlayers, thereby weakening PEO side-chain interactions with clay.	[84,86]
		Amido	Electrostatic adsorption	Amino cations electrostatically adsorb onto clay surfaces, inhibiting PEO side-chain intercalation.	[88]
Topological structure	Cross-linking agent	Cross-linked structure	Steric hindrance	The multi-arm architecture enhances steric hindrance effect, minimizing PCE molecular depletion.	[93,94,95]
		Star-shaped structure			

**Table 4:** The mechanism of different sacrificial agents.

Sacrificial Agent	Chemical Substance	Mechanism		Refs
Ionic sacrificial agent	Dimethylamine	Surface adsorption	Cationic groups adsorb electrostatically onto clay surfaces, while the terminal hydroxyl groups can further form hydrogen bonds with the clay.	[97]
	Hexadecyltrimethylammonium bromide (HTB)	Synergistic effect	HTB’s extended alkyl chain synergizes with PCE, significantly boosting its clay surface adsorption capacity.	[98]
	Alkaline electrolyte water	Cation exchange	K^+^ engages in cation exchange, while OH^−^ adsorbs onto clay particles to form an electronegative layer through surface interactions.	[99]
Non-ionic sacrificial agent	Lignin-based polyoxyethylene	Intercalation adsorption	The PEO side chain intercalates into the clay interlayer and occupies the active site.	[100]
	Small-molecule polyols	Adsorption energy	The adsorption energy between polyol and MMT exhibits an inverse correlation with the number of hydroxyl groups.	[101]
Swelling inhibitor	Ionic liquid	Surface adsorption	The cations in the ionic liquid can be adsorbed on the clay surface to inhibit the clay expansion.	[102]
	Dopamine derivatives	Stabilized clay interlayer	The protonated amine groups reduced the electrostatic repulsion between crystal layers.	[103]
	Silane coupling agent	Cation exchange	The silane modification improved the hydrophobicity of MMT and inhibited its expansion.	[104]

## Data Availability

No new data were created or analyzed in this study. Data sharing is not applicable to this article.

## Abbreviations

The following abbreviations are used in this manuscript:

PCE	Polycarboxylate superplasticizer
PEO	Polyethylene oxide
MPEG	Methoxy polyethylene glycol
APEG	Allyl polyethylene glycol
MA	Maleic anhydride
AA	Acrylic acid
HPEG	Methyl allyl polyoxyethylene ether
TPEG	Isopentenyl polyoxyethylene ether
EPEG	Ethylene-glycol monovinyl polyethylene glycol
MMT	Montmorillonite
β-CD	β-cyclodextrin
AMPS	2-acrylamyl-2-methylpropanesulfonic acid
S-PCE	Silane-modified polycarboxylic superplasticizer
PHS-PCE	Phosphonate-modified polycarboxylic superplasticizer
TPP	Polycarboxylic superplasticizer based on isopentenyl polyoxyethylene ether with introduced phosphate monomers
APC	Amphoteric polycarboxylate superplasticizer
DMC	Methylacryloxyethyl trimethyl ammonium chloride
Ct-PCE	Polycarboxylate superplasticizer with an amide cation
CPCE	Conventional polycarboxylate superplasticizer
TPCE	Polycarboxylate superplasticizer with cross-linked structure
HTB	Hexadecyltrimethylammonium bromide
AEW	Alkaline electrolyte water
PEG	Polyethylene glycol
IL	Ionic liquid
P(AM-DA)	Dopamine-derivative-grafted polymer
